# Chronic gastritis may predict risk of cerebral small vessel disease

**DOI:** 10.1186/s12876-023-03009-6

**Published:** 2023-12-07

**Authors:** Cunsheng Wei, Chang Tan, Xuemei Zhang, Xin Shen, Zongliang Xu, Junrong Li, Gelin Xu

**Affiliations:** 1 Department of Neurology, Affiliated Jiangning Hospital With Nanjing Medical University, 168 Gushan Road, Nanjing, 211100 Jiangsu China; 2 Department of Neurology, Jinling Hospital, Medical School of Nanjing University, 305 East Zhongshan Road, Nanjing, 210002 Jiangsu China

**Keywords:** Cerebral small vessel disease, Chronic atrophic gastritis, Inflammation, Stroke, Risk

## Abstract

**Background and purpose:**

Chronic gastritis, especially that caused by helicobacter pylori (HP) infection, has been associated with increased risk of ischemic stroke. But the relationship between chronic gastritis and cerebral small vessel disease (CSVD) remains largely undetermined. This study aimed to determine the potential predictors for CSVD, with chronic gastritis and its proxies as alternatives.

**Method:**

Patients aged 18 years or older with indications for electronic gastroscopy were enrolled. Presence of CSVD was evaluated with brain magnetic resonance imaging (MRI) results. Degree of CSVD was scored according to established criteria. Logistic regression analysis was used for identifying possible risk factors for CSVD.

**Results:**

Of the 1191 enrolled patients, 757 (63.6%) were identified as with, and 434 (36.4%) as without CSVD. Multivariate analysis indicated that patients with chronic atrophic gastritis had an increased risk for CSVD than those without (adjusted odds ratio = 1.58; 95% CI, 1.08–2.32; *P* < 0.05).

**Conclusions:**

Chronic atrophic gastritis is associated with the presence of CSVD. We should routinely screen the presence of CSVD for patients with chronic atrophic gastritis.

**Supplementary Information:**

The online version contains supplementary material available at 10.1186/s12876-023-03009-6.

## Introduction

The neuroimaging markers of cerebral small vessel disease (CSVD) include white matter hyperintensities (WMH), lacunes, enlarged perivascular spaces (EPVS) and cerebral microbleeds (CMBs) [1]. As a subtype of ischemic stroke, CSVD is responsible for about a fifth of stroke incidence. Coexistence of CSVD usually deteriorate stroke outcomes of other subtypes [2, 3]. CSVD is a major cause of cognitive decline and dementia in elderly, second only to Alzheimer disease [4]. But the etiology of CSVD has been far from being determined to date.

CSVD has been associated with markers of inflammation. Previous studies associated WMH and EPVS with vascular inflammation and endothelial dysfunction in stroke patients [5–7]. A cross-sectional study showed that infectious burden consisting of multiple common pathogens was associated with CMBs [8]. On the other hand, some studies failed to associate lacunes or their markers with systemic inflammation [9, 10]. Recent studies indicated that chronic gastritis, especially that caused by HP infection, were related to ischemic stroke [11–13]. HP can lead to gastric mucosal injury and other gastric diseases, both of which may enhance systemic inflammatory reaction, and, therefore, increase the risk of stroke [13]. On the other hand, HP infection may likely influence gastric physiology and absorption of micronutrients such as folate and vitamin B_12_. Deficiency of folate and vitamin B_12_ may increase serum homocysteine level and causes vascular damage [14].

Although chronic gastritis has been proved to affect the risk of stroke occurrence and recurrence, whether chronic gastritis increases the risk of CSVD is largely undetermined. This study aimed to explore the relationship between chronic gastritis and the risk of CSVD.

## Methods

### Data source

**Fig. 1:**
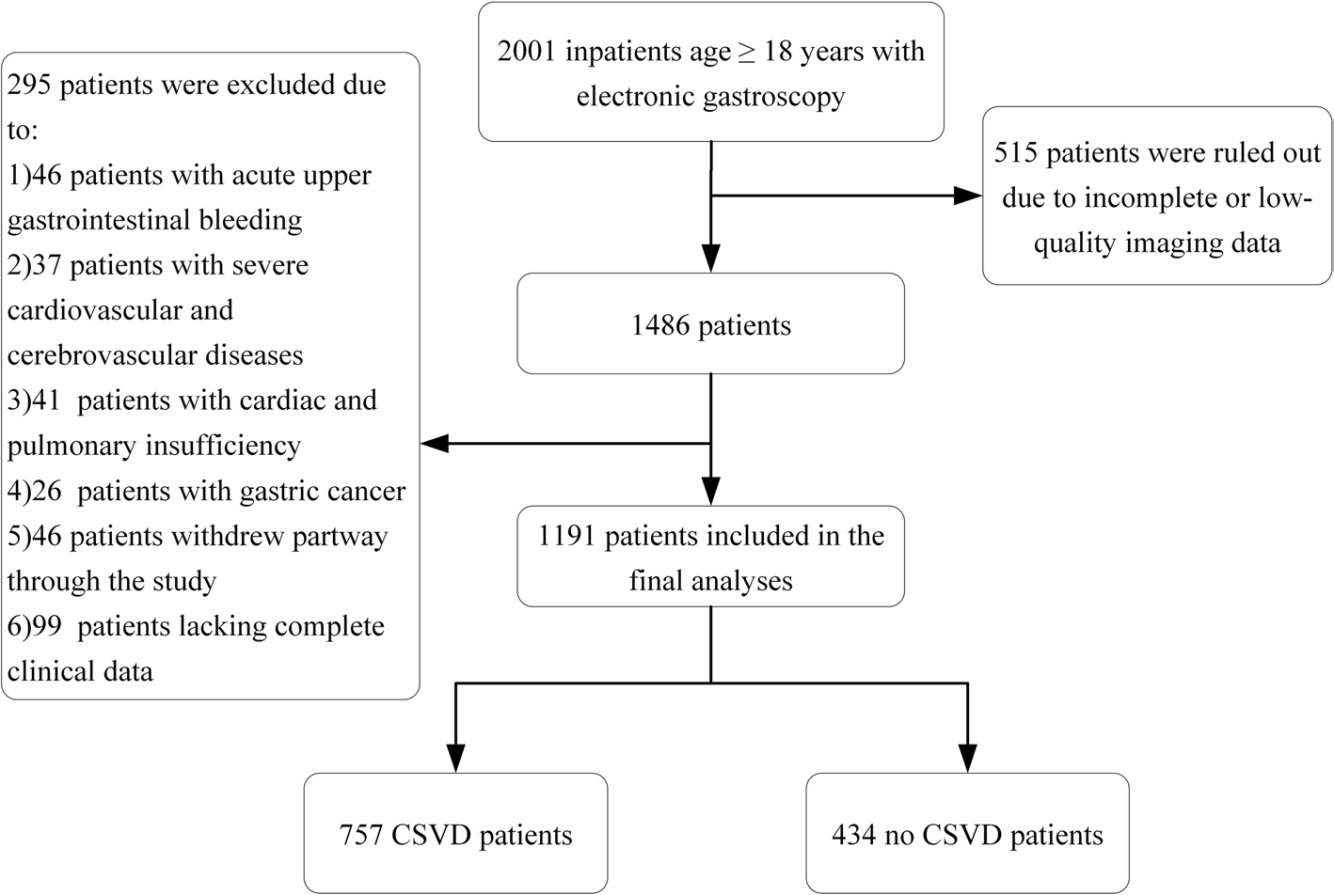
Study flowchart of patients’ selection

Patients were screened from Affiliated Jiangning Hospital with Nanjing Medical University. The present study is part of a longitudinal study on the long-term mortality of middle-aged and elderly from Jinling Hospital, Nanjing Medical University. Patients hospitalized for physical examination aged 18 years or older with indications (including gastralgia, gastric distention, nausea, vomiting, acid reflux, nausea, constipation, and diarrhea or voluntary gastroscopy) for electronic gastroscopy were enrolled from January 1, 2011 to May 18, 2020, and patients who agreed to underwent brain magnetic resonance imaging (MRI) examination within 48 h after admission were finally included in the study. Patients with acute gastrointestinal bleeding, acute cardiovascular diseases, pulmonary insufficiency, coagulation disorders and cancer were excluded (Fig. 1).

### Risk factor definitions

Risk factors were defined as the following. In this study, hypertension was defined as a blood pressure that exceeds 140/90 mm of mercury. The definition of diabetes was either being diagnosed with diabetes, or having fasting glucose levels exceeding 7.0 mmol/L. Hyperlipidemia was defined as a documented diagnosis of hyperlipidemia or on lipid-lowering medications. The presence of a previous episode of coronary heart disease or an attack of coronary heart disease at the time was considered a history of coronary heart disease. History of stroke was defined as the experience of ischemic stroke. The diagnosis of atrial fibrillation was made based on electrocardiographic evidence or self-reported physician diagnosis.

### Clinical laboratory tests

Demographic and clinical data were collected. Red blood cell counting and biochemical examinations were performed before gastroscopy examination. Presence of gastric diseases was determined according to clinical characteristics, pathological changes and gastroscopy results. Endoscopy combined with histopathological examination was used to diagnose two types of gastritis: chronic non-atrophic gastritis and chronic atrophic gastritis [15]. HP infection were determined with carbon 14 urea breath test.

### Neuroimaging evaluation

Enrolled patients underwent brain MRI examination with a 3.0 T scanner (Philips Medical Systems, Netherlands) with an 8-channel receiver array head coil. Head motion and scanner noise were reduced using foam padding and earplugs. Standardized parameters of the MRI sequences, including T1-weighted, T2-weighted and fluid-attenuated inversion recovery images were obtained. Burden of CSVD was graded as 0–4 based on imaging markers (WMH, lacunes, EPVS and CMBs) on MRI according to established criteria [16–18]. Briefly, one point represents each of the following phenomenon: more than 10 EPVS in basal ganglia, presence of lacuna, periventricular WMH with a Fazekas score of 3 or deep WMH with a Fazekas score of 2 or 3, presence of deep CMBs. Patients were then grouped as with (1–4 points) and without CSVD (0 points).

### Gastroscopy examination

Gastroscopy examination was performed with an endoscope (GIF-HQ290, GIF-H290Z; Olympus Medical Systems, Tokyo, Japan) with video processors (EVIS LUCERA ELITE CV290/CLV290SL, Olympus Medical Systems). Five gastric mucosa tissue specimens, two from gastric antrum, two from gastric body and one from gastric corner were clamped during gastroscopy examination for biopsy. Chronic inflammation, atrophy and intestinal metaplasia were diagnosed according to the Sydney system [19].

### Statistics

Continuous data were summarized as mean values with SDs for normal distribution or median value with interquartile range for skew distribution. Categorical data were presented as frequencies with proportions. Two-sample t test was used to compare continuous data. Categorical data were analyzed by the chi-square test. Logistic univariate and multivariate analyses were used for comparing group differences and identifying the risk factors of CSVD. All statistical analyses were performed using SPSS 25.0 (IBM, Armonk, NY).

## Results

Of the 1191 enrolled patients, 757 (63.6%) were identified as with, and 434 (36.4%) as without CSVD. Patients with CSVD were older than those without (58.6 ± 9.9 vs 50.2 ± 9.4; *P* < 0.001). Patients with CSVD presented lower hemoglobin concentration than those without (137.3 ± 23.8 vs 140.3 ± 16.8 g/L; *P* < 0.05). Patients with CSVD had a higher prevalence of hypertension (48.0% vs 29.5%; *P* < 0.001), diabetic mellitus (19.4% vs 11.5%; *P* < 0.001), coronary artery disease (9.8% vs 3.9%; *P* < 0.001) and chronic atrophic gastritis (21.1% vs 12.0%; *P* < 0.001) than control subjects. Conversely, patients with CSVD had a lower prevalence of hyperlipidemia (7.7% vs 22.8%; *P* < 0.001) than patients without CSVD. CSVD patients had a higher ratio of history of ischemic stroke (25.4% vs 3.0%; *P* < 0.001) than patients without (Table 1).

**Table 1:** Clinical characteristics of patients with and without CSVD

Variables	with CSVD	without CSVD	*P* value
	(*n* = 757)	(*n* = 434)	
Age, y, mean ± SD	58.6 ± 9.9	50.2 ± 9.4	< 0.001
Male, n (%)	418(55.2)	236(54.4)	0.779
Hypertension, n (%)	363(48.0)	128(29.5)	< 0.001
diabetic mellitus, n (%)	147(19.4)	50(11.5)	< 0.001
Hyperlipidemia, n (%)	58(7.7)	99(22.8)	< 0.001
Coronary artery disease, n (%)	74(9.8)	17(3.9)	< 0.001
Previous ischemic stroke, n (%)	192(25.4)	13(3.0)	< 0.001
Atrial fibrillation, n (%)	10(1.3)	1(0.2)	0.094
Cigarette smoking, n (%)	153(20.2)	89(20.5)	0.921
Alcohol drinking, n (%)	110(14.5)	67(15.4)	0.679
Presence of fecal occult blood, n (%)	127(16.8)	50(11.5)	0.045
HP infection, n (%)	282(37.3)	136(31.3)	0.088
Peptic ulcer, n (%)	162(21.4)	81(18.7)	0.283
Intestinal metaplasia, n (%)	283(37.4)	141(32.5)	0.302
Hemoglobin, g/L, mean ± SD	137.3 ± 23.8	140.3 ± 16.8	0.027
Chronic atrophic gastritis, n (%)	160(21.1)	52(12.0)	< 0.001

**Fig. 2:**
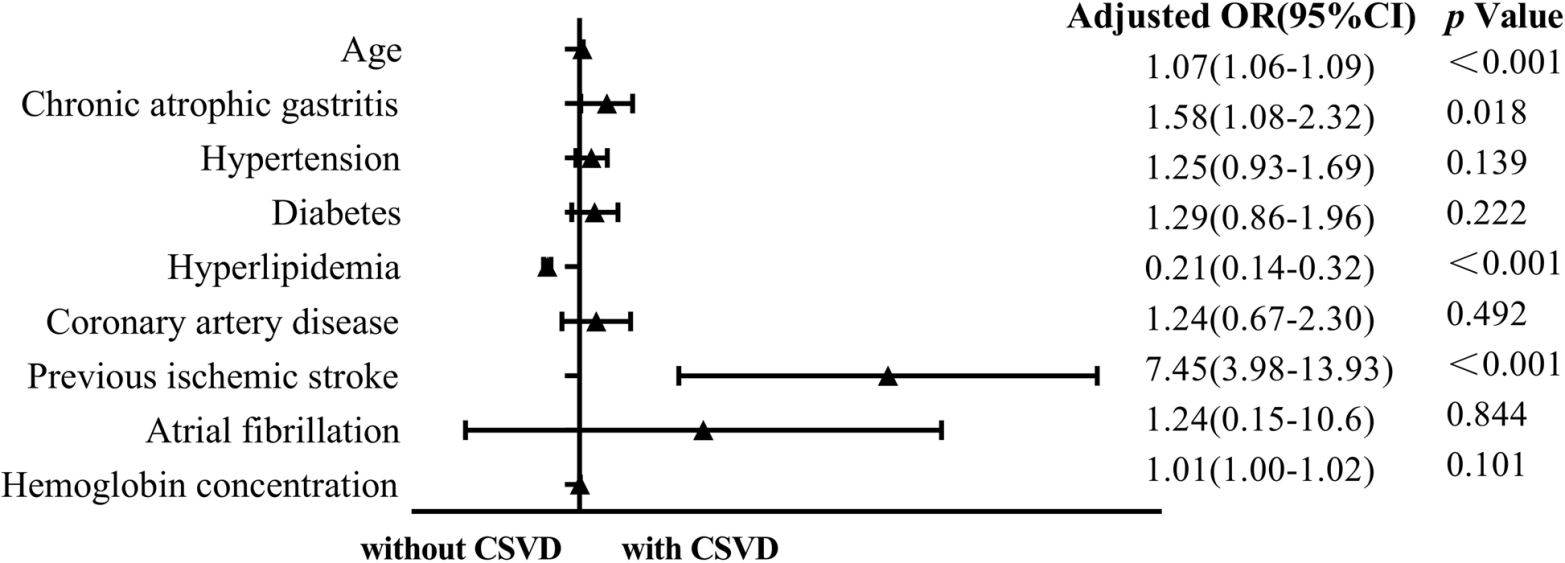
Association of chronic atrophic gastritis and CSVD presence. OR, odds ratio; CI, confidence interval. Risk of CSVD was analyzed with logistic regression models, and OR was generated. We adjusted for Age, Hypertension, Diabetes, Hyperlipidemia, Coronary artery disease, Previous ischemic stroke, Atrial fibrillation and Hemoglobin concentration in regression model 1

After adjusting for covariates, logistic regression analysis identified chronic atrophic gastritis was related to CSVD (adjusted odds ratio = 1.58; 95% CI: 1.08–2.32; *P* < 0.05). Age (adjusted odds ratio = 1.07; 95% CI: 1.06–1.09; *P* < 0.001) and previous ischemic stroke (adjusted odds ratio = 7.45; 95% CI: 3.98–13.93; *P* < 0.001) were also associated with CSVD (Fig. 2).

**Fig. 3:**
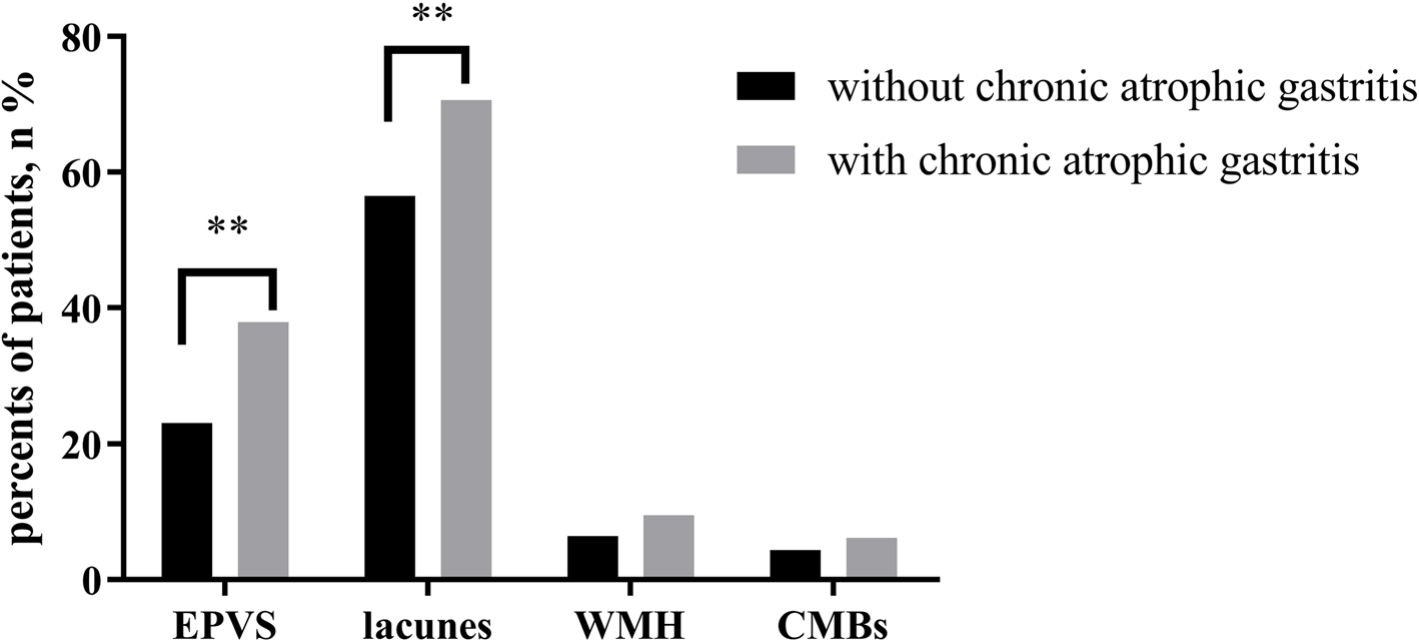
Comparison of total CSVD scores in patients with and without chronic atrophic gastritis

When patients were stratified, the results showed that patients with chronic atrophic gastritis presented significantly higher total CSVD scores than those without (1.8 ± 0.8 vs 1.5 ± 0.9, *P* < 0.01).The ratios of EPVS (37.9% vs 23.1%; *P* < 0.01) and lacunes (70.6% vs 56.5%, *P* < 0.01) in patients with chronic atrophic gastritis were significantly higher than patients without chronic atrophic gastritis. This result suggests that the difference in the total CSVD burden is mainly derived from EPVS and lacunes (Fig. 3).

## Discussion

This study found that chronic atrophic gastritis was related to CSVD and the difference in the total CSVD burden is mainly derived from EPVS and lacunes.

Compared with patients with CSVD, the proportion of hyperlipidemia in non-CSVD patients is higher, this is contrary to previous studies [20, 21]. Hyperlipidemia has been identified as a risk factor for atherosclerosis and one potential candidate risk factor for CSVD, this may be related to the intake of lipid-lowering drugs and influence of different daily life habits and activities. The traditional risk factors are only able to partially explain the presence of CSVD [22], and the incomplete understanding of the pathogenesis of CSVD limits prevention and treatment efforts. Therefore, obtaining more predictors of progression is a rational target for therapeutic treatments. In recent years, some potential new risk factors of CSVD, including chronic infection and substance abuse, have attracted the attention of scholars [23], the discovery of these new risk factors provides new thinking for the prevention and treatment of CSVD patients.

Our study revealed the higher prevalence of CSVD in patients with chronic atrophic gastritis than those without even after adjustments for multiple confounding factors. This may be related to the following factors. Firstly, chronic atrophic gastritis is an organ-specific autoimmune disease, which affects the corpus-fundus gastric mucosa [24]. The decrease or disappearance of parietal cells results in reduced or absent acid production and loss of intrinsic factor, and it further interferes with the absorption of folate and vitamin B_12_ [25], and further lead to anemia and hyperhomocysteinemia. Deficiency of folate can result in hyperhomocysteinemia, which is a possible risk factor for cardiovascular diseases [26]. One meta-analysis indicated a 10% lower risk of stroke and a 4% lower risk of overall CSVD with folate supplementation [27]. The results of the China Stroke Primary Prevention Trial (CSPPT) randomized clinical trial among adults with hypertension in China showed that folate supplementation could significantly reduce the risk of first stroke [28]. Moreover, hyperhomocysteinemia has been recognized as an important risk factor for cardiovascular diseases. It may also involve in the development of dementia, diabetes mellitus, and renal disease [29]. A controlled study showed that hyperhomocysteinemia may increase the risk of lacunar infarction and severe white matter lesion [30].

There are several limitations in the current study. First, this is an observational study without further follow-up and dynamic observation of the progress of CSVD. Second, this study is based on clinical observation. Further studies are needed to explore the possible mechanisms. Third, this study is a single-center study involving individuals in the Han population in a single center. Therefore, further multicenter studies are needed to overcome these limitations.

## Conclusions

The results of this study suggested that patients with chronic atrophic gastritis may have increased risk of CSVD. We should routinely screen the presence of CSVD for patients with chronic atrophic gastritis.

### Supplementary Information

**Additional file 1.** 

**Additional file 2.**

## Data Availability

The data that support the findings of this study are available on request from the corresponding author, upon reasonable request.

## Abbreviations

CSVD: Cerebral small vessel disease
HP: Helicobacter pylori
WMH: White matter hyperintensities
EPVS: Enlarged perivascular spaces
CMBs: Cerebral microbleeds
CSPPT: China Stroke Primary Prevention Trial
